# Cortical Bone Mapping: Measurement and Statistical Analysis of Localised Skeletal Changes

**DOI:** 10.1007/s11914-018-0475-3

**Published:** 2018-08-28

**Authors:** Graham Treece, Andrew Gee

**Affiliations:** Department of Engineering, University of Cambridge, Cambridge, CB2 1PZ UK

**Keywords:** CT, Fracture, Cortical bone mapping, Statistical parametric mapping

## Abstract

**Purpose of Review:**

Cortical bone mapping (CBM) is a technique for measuring localised skeletal changes from computed tomography (CT) images. It can provide measurements with accuracy surpassing the underlying imaging resolution. CBM can detect changes in several properties of the cortex, with no prior assumptions about the likely location of said changes. This paper summarises the theory behind CBM, discusses its strengths and limitations, and reviews some studies in which it has been applied.

**Recent Findings:**

CBM has revealed associations between fracture risk and cortical properties in specific regions of the proximal femur which present feasible therapeutic targets. Analyses of several pharmaceutical and exercise interventions quantify effects that are distinct both in location and in the nature of the micro-architectural changes. CBM has illuminated age-related changes in the proximal femur and has recently been applied to other bones, as well as to the assessment of cartilage.

**Summary:**

The CBM processing pipeline is designed primarily for large cohort studies. Its main impact thus far has not been in the realm of clinical practice, but rather to improve our fundamental understanding of localised bone structure and changes.

## Introduction

Imaging plays an important role in the investigation and understanding of skeletal disease. The most commonly used modality for osteoporosis assessment is dual-energy X-ray absorptiometry (DXA), since it is relatively safe, well understood, and capable of measuring properties that are good predictors of fracture [1]. Nevertheless, there are advantages to be gained through the use of modalities, such as quantitative computed tomography (QCT), that are able to reveal the 3D structure of the bone. QCT is an essentially conventional CT with an additional calibration phantom for converting Hounsfield units to material densities. Although even high-resolution peripheral QCT (HRpQCT) cannot delineate precisely the micro-architecture of cortical and trabecular bone [2], QCT can capture far more detail than is possible with DXA. A number of emergent techniques have been proposed for the analysis of skeletal QCT data [3], motivated by the need to further understand the links between bone health, fracture risk, and the specific benefits of pharmaceutical treatments and exercise regimes. Early detection and improved diagnostic accuracy are important goals, since there can be a latency of several years before treatments start to show any benefit [4].

Traditionally, QCT studies involved specific types of image analysis within predetermined regions of interest, for instance the average bone density within the femoral neck. In contrast, computational anatomy techniques [3, 5] permit the study of each bone as a whole, looking for effects over the entire volume or surface without preconceptions regarding their location or nature. The discipline of computational anatomy encompasses mechanical assessment of bone strength (finite element analysis, FEA), volumetric image analysis (voxel- and tensor-based morphometry, VBM or TBM) and surface-based image analysis (cortical bone mapping, CBM). While CBM is the focus of this review, the statistical approaches required to quantify effects in CBM are similar to those needed for VBM and TBM, and the accurate cortical measurements that underpin CBM may also be used to improve the quality of FEA bone models.

CBM studies have demonstrated links between bone features and ageing, fracture risk, and even radiotherapy, and also the effects of pharmaceutical and exercise regimes. Since CBM is surface-based, results are best displayed as colour maps, revealing where on the bone surface the effects are most prominent. The source data is typically whole-body QCT [3, 6•], though HRpQCT has been used for studies of the distal limbs [7] and palaeoanthropological specimens [8]. However, neither HRpQCT nor QCT can reveal the porous structure of bone, since the imaging resolution is limited to around 0.3 mm for the former and 1.5 mm for the latter. Much of the CBM pipeline is therefore concerned with making accurate measurements of cortical properties in low-resolution data. Further challenges are posed by the sheer quantity of measurements (thousands per scan, with typically hundreds of scans per study) and their subsequent statistical analysis. It is therefore welcome that free software is available for each stage of the CBM pipeline.^1^

## Technical Overview

**Fig. 1:**
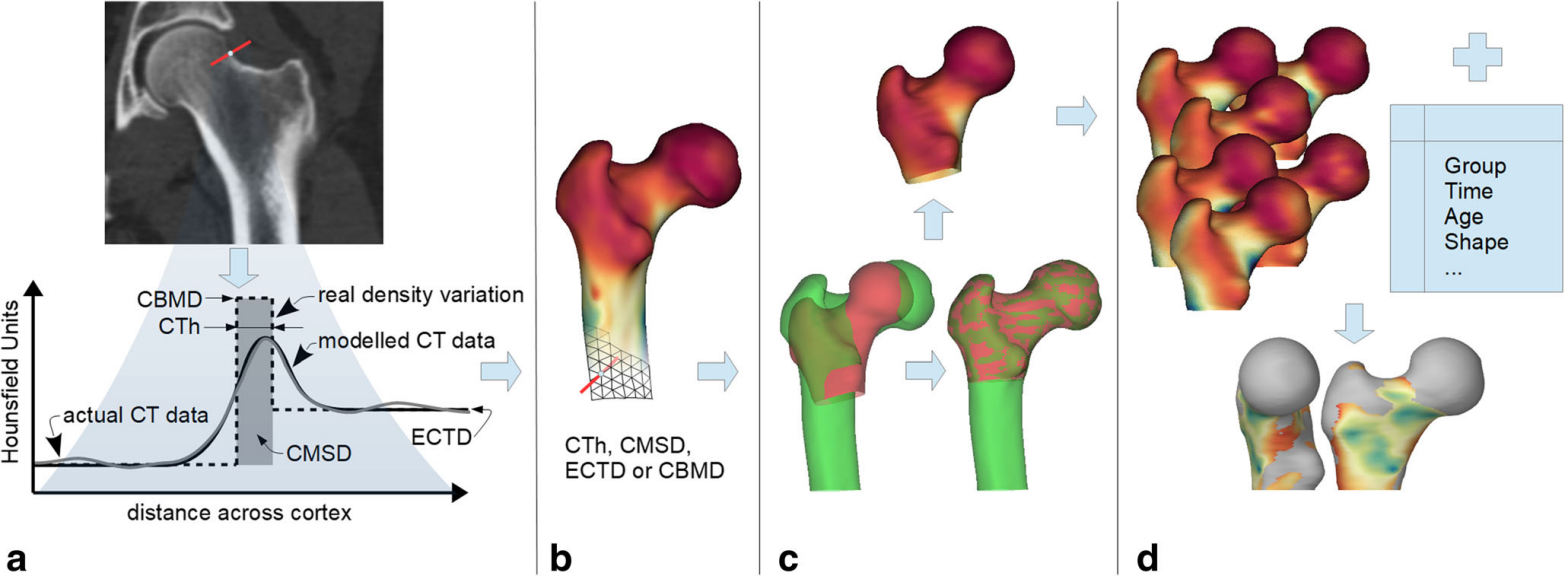
The cortical bone mapping (CBM) pipeline. **a** Bone properties at a particular location are measured from CT data sampled on a line passing at right angles through the cortical surface. The key measurements are of cortical thickness (CTh, mm), cortical bone mineral density (CBMD, mg/cm^3^), endocortical trabecular density (ECTD, mg/cm^3^), and cortical mass surface density (CMSD, mg/cm^2^), all but CTh requiring the presence of a calibration phantom for conversion from Hounsfield Units. **b** The measurements are repeated at many locations over the surface of the bone, with the location and direction of each measurement guided by an approximate segmentation of the periosteal surface. Cortical properties can be visualised on this surface by mapping to a range of colours. **c** In order to compare properties over bones from multiple subjects or at multiple time points, each surface is aligned with a template (canonical) surface. The individual sets of cortical data are then transferred onto the canonical surface. **d** The mapped data from all subjects or time points is then considered alongside potential regressors, which are typically demographic and study data (e.g. age, weight, time point in a longitudinal study, case or control group in a transverse study, shape). Statistical parametric mapping (SPM) is used to identify regions on the surface where the cortical properties depend significantly on the various regressors

CBM is a procedure for making accurate measurements of cortical and endocortical trabecular quantities, distributed over the surface of a bone, from CT data of many different subjects and perhaps at different time points, and combining the measurements to demonstrate statistically significant effects across the cohort [6•, 9, 10]. CBM can hence be used to answer questions such as “Does this therapy change the thickness or density of bone, by how much, and where on the bone surface is the effect significant?” or “How does the distribution of bone change with age?” The CBM pipeline is outlined in Fig. 1, with the various stages summarised in the caption and described in more detail below.

### Surface-Based Bone Measurement

CT imaging systems have finite resolution, and hence each CT sample reflects the X-ray linear attenuation of the imaged material averaged over a small volume. This attenuation is usually expressed in Hounsfield units (HU), where -1000 HU corresponds to air and 0 HU to water. Despite this normalisation, HU values for tissue and bone depend on the energy distribution of the X-rays, which varies between CT scanners. In QCT, a calibration phantom is scanned to facilitate conversion from HU to bone-equivalent material density (BMD, mg/cm^3^). The finite resolution volumes are generally larger than the voxel size (the spacing between samples in the 3D data set). In whole-body CT, the volumes’ in-slice dimension is typically 1.5 mm, with the between-slice dimension depending on slice spacing, but typically exceeding the in-slice dimension.

For structures that are larger than the finite resolution volume, the effect is to blur their edges a little, with no catastrophic consequences for thickness or density measurement. However, trabecular bone, and cortical bone in many places, is thinner than the finite resolution volume, and consequently QCT measurements may not be what they seem [11]. It is quite possible for an apparent measurement of cortical ‘density’ to be more representative of cortical ‘mass’, and for cortical ‘thickness’ to reflect the imaging resolution above any property of the cortex itself. Much depends on how the measurements are taken, a detail which is frequently lacking in the literature, making inter-study comparisons very difficult.

**Fig. 2:**
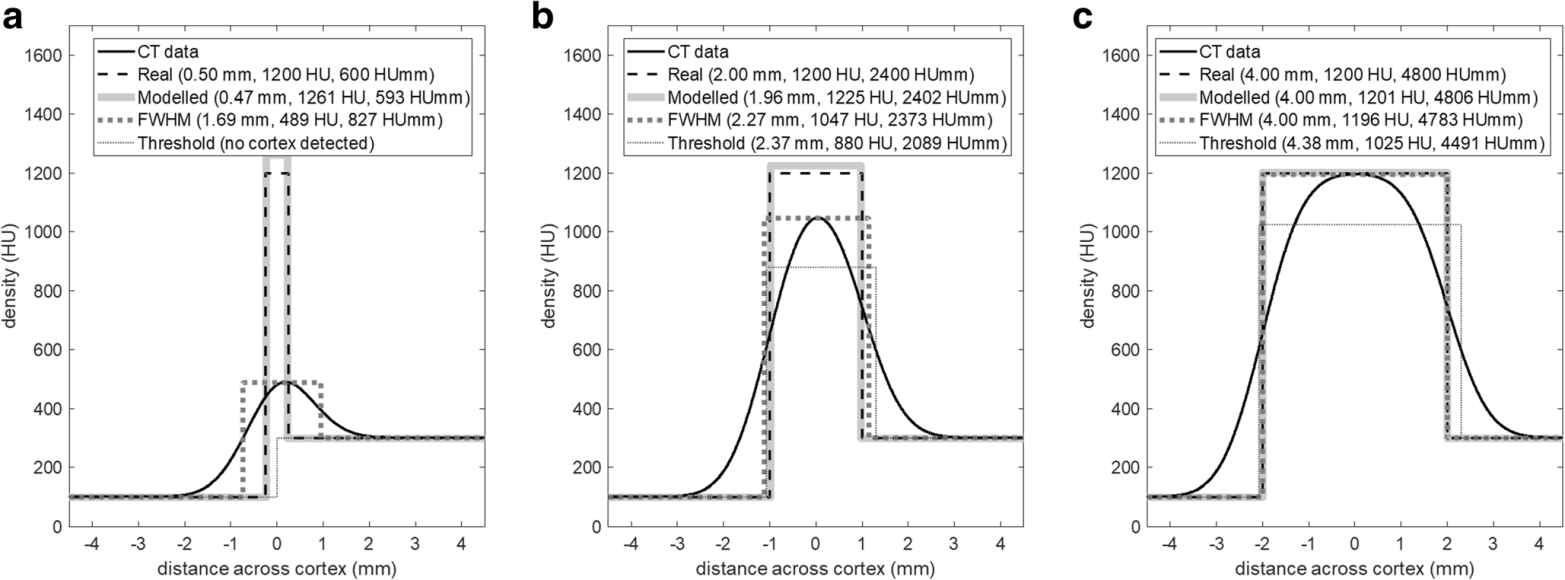
Cortical measurements. For high-resolution data (e.g. peripheral HRpQCT), thresholding, model-based, and full-width half-maximum (FWHM) techniques can all be successful. However, for low-resolution, clinical QCT, a model-based approach (as in Fig. 1a) is preferred. In such an approach, the density variations through the cortex, and the imaging blur, are varied until they are consistent with the observed CT data. Three cases are illustrated here, all with typical QCT blurring. The actual density through the cortex is shown dashed, with corresponding CT data as a solid line. The lighter grey lines are the model-based, thresholded, and FWHM results. **a** A very thin cortex (typically *<* 1 mm) for which thresholding misses the cortex entirely, FWHM results in considerable overestimation of CTh and underestimation of CBMD, and the model-based method is more accurate. **b** A thicker cortex (typically between 1 and 3 mm) for which the model-based estimate is substantially correct, FWHM slightly overestimates CTh and underestimates CBMD, and the accuracy of thresholding depends entirely on the selected threshold. **c** For thick cortices (typically *>* 3 mm), FWHM and model-based techniques are accurate, but thresholding may underestimate CBMD if the CT data is averaged over the cortex

With CBM, the aim is to measure cortical and endocortical trabecular properties that are as faithful as possible to the underlying physical quantities, even for very thin cortices that are well within the resolution of the CT scanner. Figure 2 shows three examples of simulated CT data that illustrate how CBM differs from other techniques. The graphs show CT values on a line passing perpendicularly through the cortex, which is assumed to have a density of 1200 HU, surrounded by soft tissue of 100 HU and trabecular bone with average density 300 HU. Only the cortical thickness differs between the three examples. While the true density (dark dashed line) changes abruptly at material boundaries, the measured CT values (dark solid line) are averaged within the finite resolution volume and therefore change more gradually.

Thresholding is the most common technique for estimating cortical thickness and density. Cortical bone is detected when the CT data exceeds a predetermined threshold; cortical thickness follows as the distance between points where the CT data crosses the threshold, and cortical density is the average of the CT values between these points. Alternatively, the threshold may be adapted to local properties of the data. If it is set to halfway between the peak and surrounding CT values, we have the full width half-maximum (FWHM) technique [12], with the density then given by the peak value. In contrast, CBM is a model-based approach. The various model parameters (material densities, cortical edge locations and the CT blur) are initially guessed, the resulting CT data is simulated and compared with the actual CT data, and the model parameters are then optimised until the simulated and real data match. This approach was initially proposed for the measurement of cartilage [13] and later adapted for cortical bone analysis [9]. CBM was further developed to improve density estimation [6•, 10] and is now widely adopted by the research community [14–17].

Inspection of the legends in Fig. 2 shows that FWHM and thresholding produce poor estimates of thickness (mm) and ‘density’ (HU), whereas CBM is more reliable, especially when the cortex is thin. There are some subtleties not discussed here, particularly when it comes to density estimation which, for very thin cortices, is inherently less precise than thickness estimation [6•, 10]. However, the broad conclusions remain unchanged, with CBM consistently outperforming the other techniques.

While cortical thickness (CTh, mm) and ‘density’ (in HU) can be measured without a calibration phantom, QCT data is required for true cortical bone mineral density (CBMD, mg/cm^3^). However, the most reliable measurement that can be made is cortical mass surface density (CMSD, mg/cm^2^). This is a poorly understood quantity, but of significant value precisely because it can be measured with high accuracy and is a good indicator of cortical bone strength. Unlike the DXA-based quantity ‘areal density’ (aBMD), which has the same units but is not a true measure of density at all, CMSD is a well-defined material property, the cortical mass per unit surface area, calculated by multiplying CBMD × CTh. Since either thicker or denser bone result in increased CMSD, CMSD is a good indicator of bone strength. The legends in Fig. 2 show uncalibrated CMSD in units of HUmm. Note how CBM consistently estimates CMSD with high accuracy, even when the cortex is thin.

Unlike VBM and TBM, CBM is intrinsically a surface-based technique designed to estimate properties of cortical bone. Nevertheless, the model-fitting produces an estimate of the average trabecular density immediately adjacent to the cortex, the endocortical trabecular density (ECTD, mg/cm^3^). Endocortical trabecular bone supports the cortex and therefore plays an important role in bone strength. In total, then, CBM produces four measurements which can be mapped across the surface and analysed for statistically significant effects: CTh, CBMD, CMSD, and ECTD. These four measurements are shown in Fig. 1a.

### Mapping of Measurements

Having established how to measure cortical properties at a single location, the next step is to repeat this process at many locations covering the region of interest on the bone. This requires an approximate representation of the bone surface as a *triangle mesh*, a set of connected triangles which is the standard way to represent surfaces in computer graphics. Part of the underlying triangle mesh is revealed in Fig. 1b. Cortical measurements are then made at each vertex of the mesh, in the direction of the normal to the mesh at that vertex. The measurements are not particularly sensitive to the form or location of the mesh, provided the vertices are within around 2 mm of the true cortical surface [1]. In most CBM studies, the mesh is generated by a semi-automatic segmentation technique, taking around 10 min per proximal femur [6•]; more automated methods are also possible [14]. An even distribution of triangles, as shown in Fig. 1b, is necessary for regular sampling of the cortical properties on the surface.

CBM measurements may then be displayed as colours on the mesh. Variations in hue are essential for this purpose, since brightness is already exploited to create the 3D impression. The choice of colour scale has a strong impact on perception of the measurements. A good scale should not exhibit mach banding (abrupt, perceived transitions at arbitrary measurement values) and should reveal small variations at all ranges [18]. The ubiquitous ‘rainbow’ colour scale fails on both these counts, while the scale used throughout this paper is sound.

### Registration and Statistical Parametric Mapping

In typical studies, cortical maps are produced for hundreds of individuals and then analysed using statistical parametric mapping (SPM), a methodology that has its roots in neuroimaging [19]. A prerequisite, termed *spatial normalisation* in the SPM lexicon, is that the cortical measurements are transferred onto a template (or canonical) surface. This is achieved by aligning the canonical surface with each individual, and then projecting the measurements from the vertices of the individual mesh onto the nearest vertices on the canonical mesh, as shown in Fig. 1c.

Surface-to-surface alignment (or *registration*) is a common procedure in medical image analysis. Registration algorithms lie on a spectrum from fully automatic to highly manual, the latter requiring expert labelling of anatomical landmarks. While the automatic methods have obvious appeal, the registrations they produce can be somewhat arbitrary. Thus, individuals with identical cortical properties, but different shapes, may align differently with the canonical surface, and the cortical properties will no longer appear identical on the canonical mesh [20]. Systematic misregistration of this nature is particularly problematic when the study explicitly references shape (e.g. “How does CMSD depend on femoral bone shape?”), in which case registration based on explicit anatomical landmarks is preferred [21]. A by-product of the registration process is a compact representation of each specimen’s shape [22], which may be of interest in its own right or incorporated as a regressor in the subsequent statistical analysis.

Finally, the cortical measurements, now all expressed on the canonical mesh, are smoothed before a general linear model (GLM) is fitted to the data at each vertex. For example, a case-control study might fit a model in which CMSD is explained by age, weight, shape, and group (case or control). *F* or *t* statistics are then calculated at each vertex, to test whether CMSD depends significantly on the regressors, with random field theory furnishing the corresponding *p* values, corrected for multiple comparisons to control the overall image-wise chance of false positives. The coefficients of the GLM can be masked to highlight those regions where the effect is statistically significant, for example with *p <* 0*.*05, as shown in Fig. 1d. Alternatives to SPM include principal component analysis (PCA) of the cortical data, with *t* tests to detect significant differences in PCA coefficients between groups [23].

## Validation

The accuracy of the first stage of CBM has been assessed by comparing measurements from low-resolution, QCT scans of cadaveric femurs with measurements of the same femurs obtained with higher-resolution, HRpQCT imaging at 82 μm. The initial CBM study [9] assessed 16 specimens from the Melbourne femur collection, of mixed sex and ages 40–83, revealing CTh accuracy of 0*.*0 ± 0*.*6 mm for cortices in the range 0.3–4 mm. Follow-on studies, with improved methodology and a larger cohort of 18 female and 17 male specimens of ages 59–96 years from the Medical University of Vienna [6•], demonstrated CTh accuracy of 0*.*1 ± 0*.*4 mm (for the range 1–6 mm) and 0*.*2 ± 0*.*2 mm (for 0.3–1 mm), CBMD accuracy of − 30 ± 180 mg/cm^3^ (for 1–6 mm) and 190 ± 330 mg/cm^3^ (for 0.3–1 mm), CMSD accuracy of 8 ± 25 mg/cm^2^ (for 1–6 mm) and 1 ± 11 mg/cm^3^ (for 0.3–1 mm), and ECTD accuracy of − 20 ± 60 mg/cm^3^ (for 1–6 mm) and 3 ± 30 mg/cm^3^ (for 0.3–1 mm). Similar validation methods for CBM measurements of the skull assessed CTh accuracy as 0*.*1 ± 0*.*6 mm for the range 0–4 mm, despite complications caused by the close proximity of the inner and outer tables [24].

**Fig. 3:**
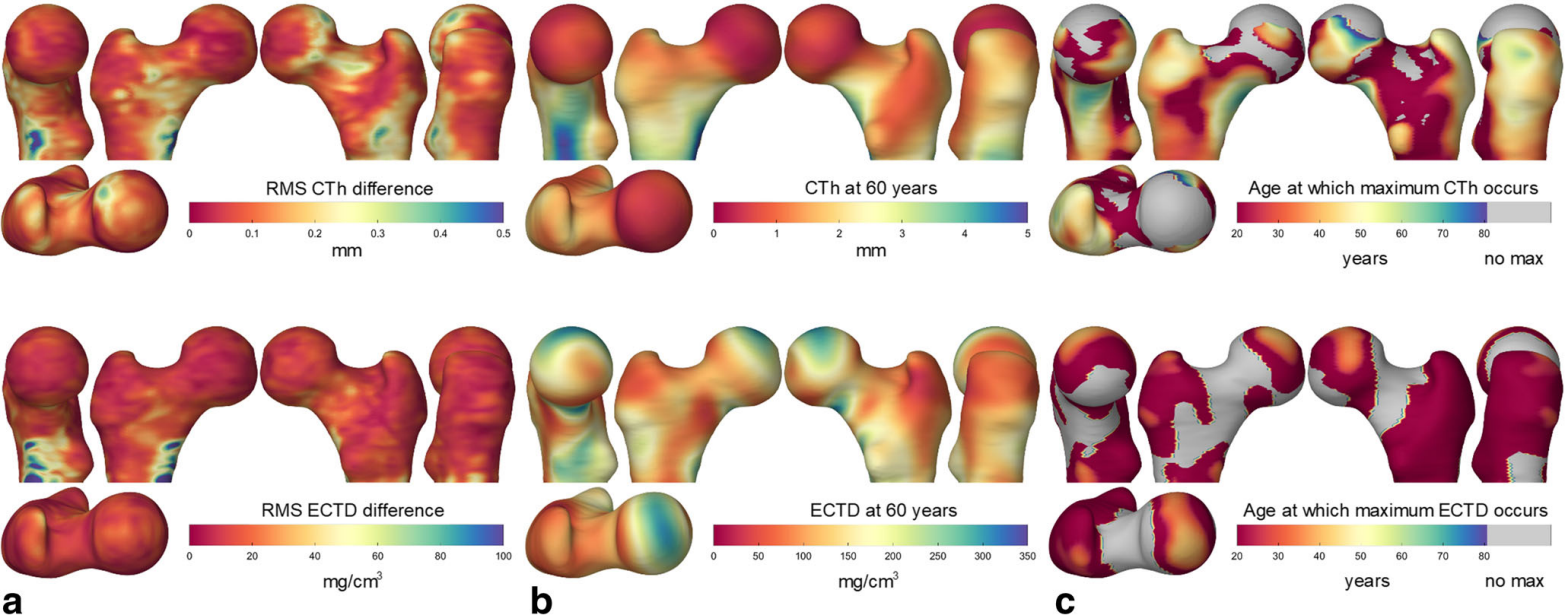
Typical CBM measurement errors and CBM distribution with age. **a** The RMS error of each local CTh (top) and ECTD (bottom) measurement is estimated by repeat scanning of a number of subjects, within 3 months, over which time only small changes would be expected. **b** Typical CTh and ECTD for a 60-year-old female, bone health being of particular interest in the elderly female population. **c** Across much of the proximal femur, CTh and ECTD peak and then start to decrease at a certain age, but that age varies with location. For a female cohort, CBM analysis reveals the peak age, displayed here as a colour map, with no significant peak detectable in the areas shaded grey

These results should be placed in context through coefficients of variation (CV), which express measurement precision relative to the population-wide variation of the quantity being measured. For individual measurements, CV is 4% for CTh based on immediate repeat scanning [14], or 6% for CTh, 3% for CBMD, 5% for CMSD, and 9% for ECTD based on repeat scanning after 3 months [1] (see Fig. 3a). When averaging results over regions of interest for fracture prediction, the CVs reduce to less than 1% for all but CBMD (2%).

## Applications

Since the *raison d’etre* of CBM is to reveal the location as well as the significance of skeletal changes, CBM results are best summarised pictorially. We therefore provide a number of figures which are collated and normalised to assist in inter-study comparisons. When the sample size is small, it is sometimes necessary to average cortical measurements over the surface to arrive at statistically significant results. Any differences in the CTh, CMSD, ECTD and CBMD effects provide insight into the mechanisms of cortical change.

### Proximal Femur

CBM was used to quantify healthy ageing in a cohort of 619 Caucasian women aged 19–97 [25]. The results provide a standard reference for each of the CBM measurements, and highlight how different areas of the proximal femur are preserved into later life. Figure 3b, c demonstrates that, while ECTD starts to deteriorate at most locations from an early age, CMSD and CTh are preserved in some areas well into the sixth decade [25].

**Fig. 4:**
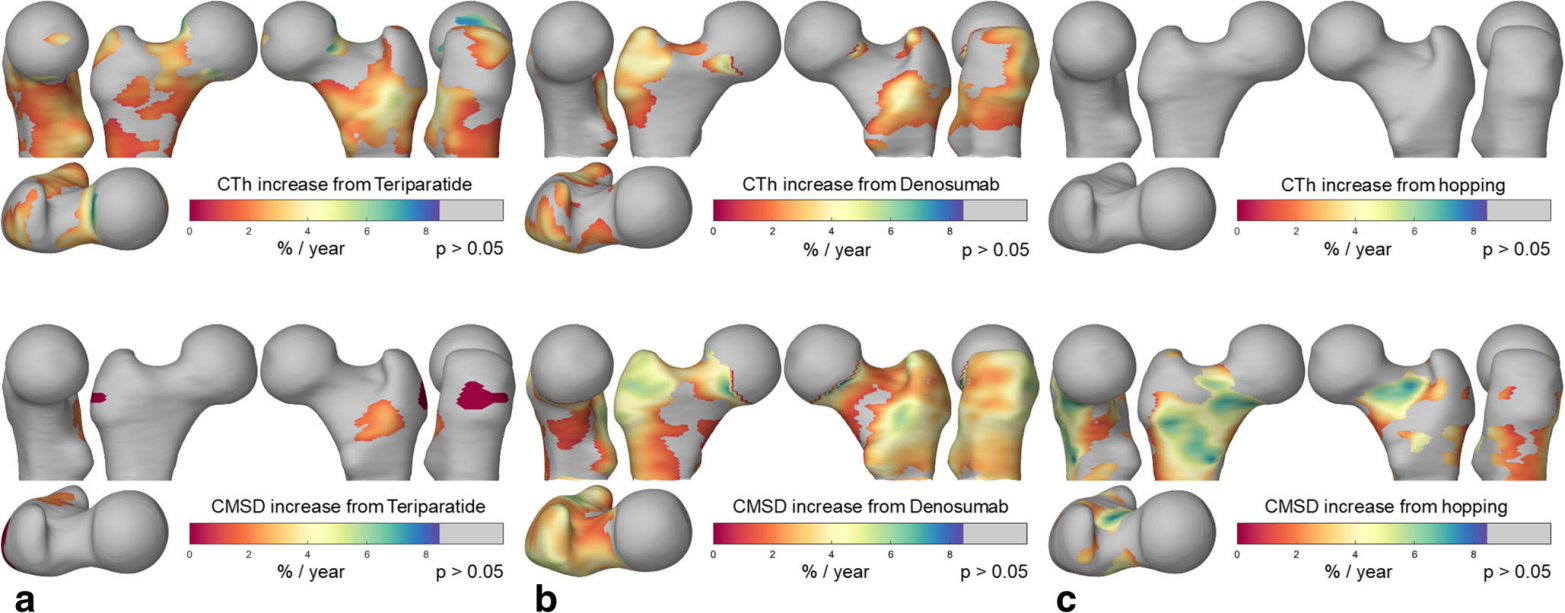
The effects of teriparatide, denosumab, and an exercise regime. All figures show percentage increases per year, with grey indicating no significant change, and consistent colour scales to facilitate direct comparisons. Although the magnitude of the effects is similar across all three studies, the location and nature of the effects are markedly different. **a** Teriparatide provokes an increase in CTh (top), but much less so CMSD (bottom), which implies that there has either been a decrease in cortical density or an increase in porosity. **b** Treatment with denosumab increases both CTh and CMSD, though more so the latter, implying that the dominant effect is reduced porosity or increased cortical density. **c** An exercise regime, involving regular hopping on one leg, provokes a comparable increase in CMSD of the exercise leg, but not CTh, again signifying more dense or less porous bone

CBM has also been used to assess a number of therapies. Figure 4 compares three of these, with effects shown on the same scale and normalised to a single year period. The teriparatide (TPTD) study analysed 119 femurs from 65 women of mean age 68, over 24 months of treatment [26]. Response to denosumab was measured for 80 female subjects, aged 60–90, at baseline and then after 1, 2, and 3 years of treatment [27]. The exercise regime was assessed over a period of 1 year for a cohort of 34 men with an average age of 70 [28, 29•]. Although all three interventions produce effects of similar magnitude, Fig. 4 reveals significant differences in the locations of the effects, and in the degree to which the interventions target CTh as opposed to CMSD. Exercise-induced changes have also been recorded using VBM [30].

A further CBM study measured the effects of switching treatment from alendronate (ALN) to TPTD, compared with adding TPTD to ongoing ALN [31]. The results demonstrated more significant increases in CTh and decreases in CBMD when switching from ALN to TPTD, suggesting that ALN moderates the effects of TPTD, particularly in load-bearing regions. In another multi-trial analysis, the effects of TPTD over different treatment periods of 18 and 24 months were compared [32]. Despite difficulties analysing data over multiple trials, the results showed a statistically significant increase in ECTD from 18 to 24 months, confirming continued TPTD effects over the longer treatment period.

Several studies have used CBM to identify links between focal cortical defects and fracture risk. The first such study combined data from the Czech Republic and the UK, looking at 313 female and 30 male subjects, with 158 fractures, 145 controls, and 50 fallers without fracture [33•]. The results revealed distinct patterns of CMSD and ECTD linked to different fracture types, with focal reductions of around 20% CMSD and 50% ECTD in cases compared with controls. It was further demonstrated that CBM predicts fracture type better than aBMD measurements alone.

**Fig. 5:**
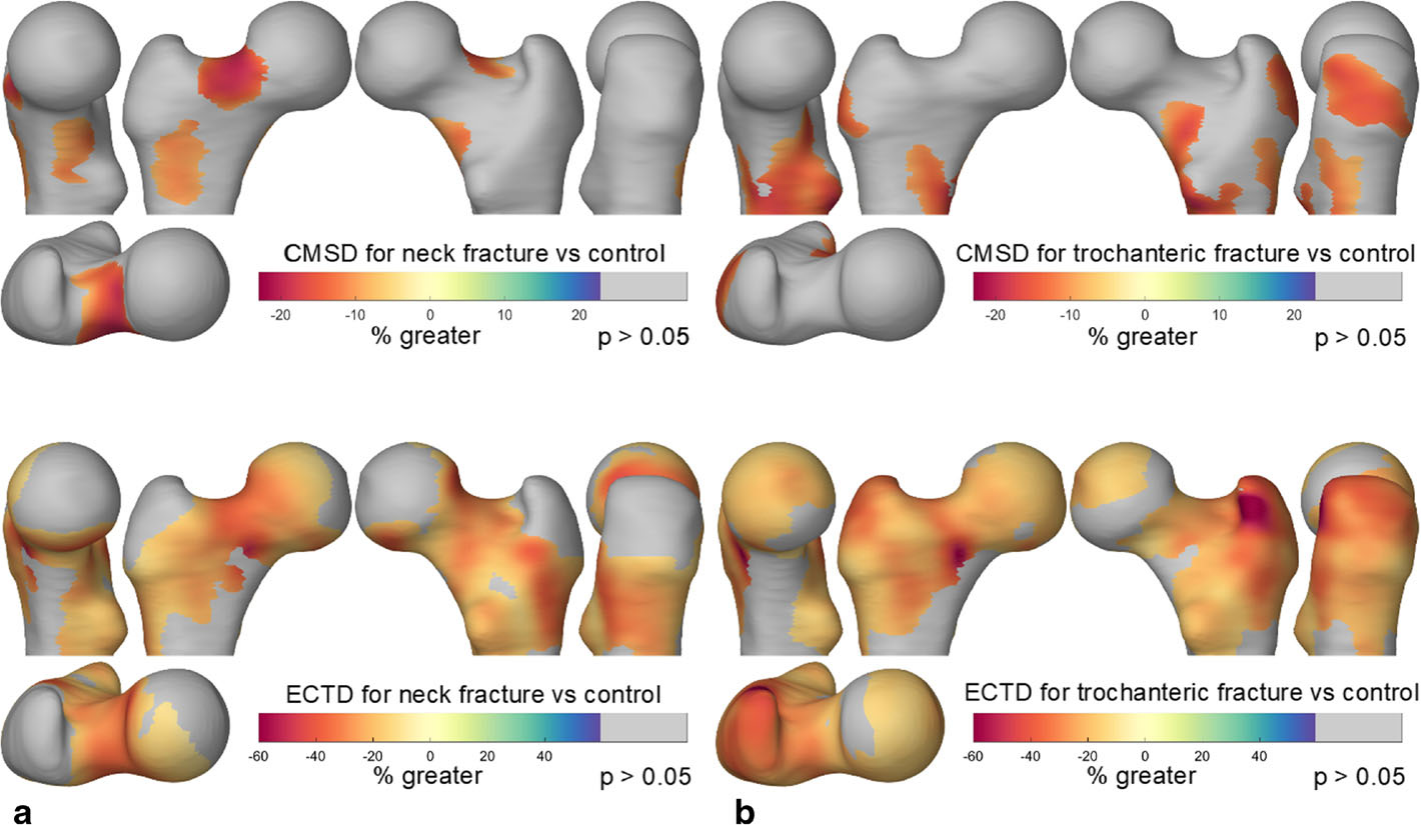
Analysis of fracture risk. The figures show areas of the cortex that are implicated in two types of fracture, based on data from a prospective study of several hundred males. **a** CMSD (top) and ECTD (bottom) linked to femoral neck (cervical) fracture. There is a highly significant patch at the superior femoral neck where CMSD is 20% lower in cases than in the wider cohort. In the same region, ECTD is 40% lower in cases than in the cohort. **b** Areas of the cortex associated with trochanteric fracture are more diffuse, but note the peak deficits at the greater trochanter

The second study, which is presented in Fig. 5, was a prospective study of 99 male cases alongside a cohort of 308 males, with 44 trochanteric and 55 cervical fractures [1]. This revealed similar focal defects to the aforementioned Czech/UK study. When predicting fracture type (cervical or trochanteric), the area under the receiver operator characteristic for trochanteric fractures increased from 0.71 (based on DXA-derived parameters) to 0.77 (including CBM parameters). For cervical fractures, the increase was from 0.76 to 0.82. Adding CBM to DXA-derived measurements led to a small but significant improvement in combined fracture prediction, whereas adding DXA to CBM made no difference. A more recent CBM investigation of fracture prediction revealed similar focal defects [34], as did a previous study using VBM [35]. Interestingly, the defect at the superior femoral neck, strongly associated with cervical fracture, appears to be more prevalent in individuals with larger bones [36].

CBM of the proximal femur has value beyond treatment monitoring and fracture prediction. For example, CBM-based thickness and density measurements have been used to build FEA models that allow sophisticated assessment of bone strength in relation to fracture risk [37, 38]. CBM has also been used to monitor adverse effects of radiotherapy in patients with anal cancer. In [39], the hips of 22 patients were scanned at baseline and after radiotherapy. The pre-therapy and post-therapy hips were aligned using anatomical landmarks and then compared at several a priori regions of interest. The results showed that a dose exceeding 40 Gy was a significant predictor of clinically significant focal cortical bone thinning, with *>* 30% reduction in CTh at the femoral neck and a 6% reduction in ECTD as well.

### Other Skeletal Sites

**Fig. 6:**
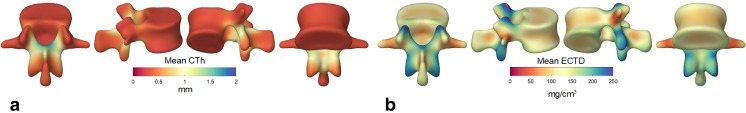
CBM applied to the L1 vertebra. Mean CTh (**a**) and ECTD (**b**) maps are shown for a cohort of women aged 66 years on average

Figure 6 shows baseline CTh and ECTD of the L1 vertebra in a study of 56 women (mean age 66), from a trial investigating the effects of romosozumab (ROMO) and TPTD administered over the course of 1 year [40]. Compared to baseline, TPTD increased CTh by 6%, ECTD by 17%, and CMSD by 5%, whereas ROMO increased CTh by 12%, ECTD by 22% and CMSD by 13%, with the ROMO effects significant across most of the vertebral body. Age-related bone loss at the vertebrae has been assessed using VBM [41].

CBM has also been used to investigate links between thoracic stereotactic radiotherapy and spontaneous fracture of the ribs [42]. The methodology was similar to the aforementioned femoral radiation study [39], with a priori regions of interest in lieu of SPM. In a cohort of 28 patients, there was a clear relationship between CTh and dose, with 2% CTh thinning for doses of 0–10 Gy, 7% for 10–20 Gy, 14% for 20–30 Gy, 15% for 30–40 Gy, and 18% for *>* 40 Gy. Healthy variation in cortical thickness of the ribs has been studied in a similar manner [43].

CBM has furthered understanding of injury biomechanics by providing more detailed measurements of skull cortices in healthy ageing [44], though again the statistics were handled differently and not using SPM.

Finally, CBM has found application in the field of palaeoanthropology. Although sample sizes tend to be small, and statistical power is therefore diminished, CBM studies of the hand and foot bones of extant and extinct hominoids have shed light on skeletal loading and function [8, 45].

## Conclusions and Future Directions

CBM is a powerful methodology that offers accurate assessment of the skeletal cortex from low-resolution QCT data, with good differentiation between thickness, density, and mass. By transferring the measurements onto a canonical surface, and analysing the resulting distributions using SPM, it is possible to identify regions where the cortical properties depend on regressors of interest (for example age, or case/control, or baseline/post-treatment), with no need for a priori assumptions as to the locations of said regions. Weaknesses of CBM include the need for an approximate segmentation of the outer bone surface, which is typically the most time-consuming stage of the processing pipeline. Unlike the voxel-based alternatives VBM and TBM, CBM provides information only about the cortex and the endocortical trabecular bone.

Two recent developments indicate likely future trends in CBM-type analysis. By extending the cortical model to allow for a finite-width, endocortical region where the bone density decreases linearly from cortical to trabecular levels, it is possible to detect and assess specifically endocortical bone remodelling. It has recently been demonstrated that such measurements are feasible using low-resolution, clinical QCT [46]. Secondly, since CBM is fundamentally concerned with the measurement of thin plate-like structures, it can be applied not just to the skeletal cortex, but to any other thin structure. In this spirit, CBM has been used to assess the space between bones in a joint [47], which is indicative of cartilage thickness and therefore important in the evaluation of osteoarthritis. Whereas cartilage can only be inferred indirectly from the joint space in CT, CBM has also been successful in assessing cartilage thickness directly, at the knee, using MRI [48].

## Footnotes

1 Stradwin: http://mi.eng.cam.ac.uk/~rwp/stradwin/ wxRegSurf: http://mi.eng.cam.ac.uk/~ahg/wxRegSurf/ Surfstat: http://www.math.mcgill.ca/keith/surfstat/
